# Supplementary material for: The Role of Cell Wall Polysaccharides Disassembly and Enzyme Activity Changes in the Softening Process of Hami Melon (Cucumis melo L.)
Source: Foods. 2022 Mar 15;11(6):841. doi: 10.3390/foods11060841 (PMC8954864; doi:10.3390/foods11060841)
Supplement: Supplementary file 1 [file foods-11-00841-s001.zip › foods-1632435-supplementary.pdf]

foods

MDPI

Article

The Role of Cell Wall Polysaccharides Disassembly and Enzyme Activity Changes in the Softening Process of Hami Melon (*Cucumis melo* L.)

Weida Zhang, Minrui Guo, Wanting Yang, Yuxing Liu, Yue Wang and Guogang Chen *

College of Food Science and Technology, Shihezi University, Shihezi 832000, China; zwd9411@163.com (W.Z.); gmrshzu@163.com (M.G.); 18139280260@163.com (W.Y.); liuyuxing233@163.com (Y.L.); 17590396006@163.com (Y.W.)

* Correspondence: cgg611@163.com

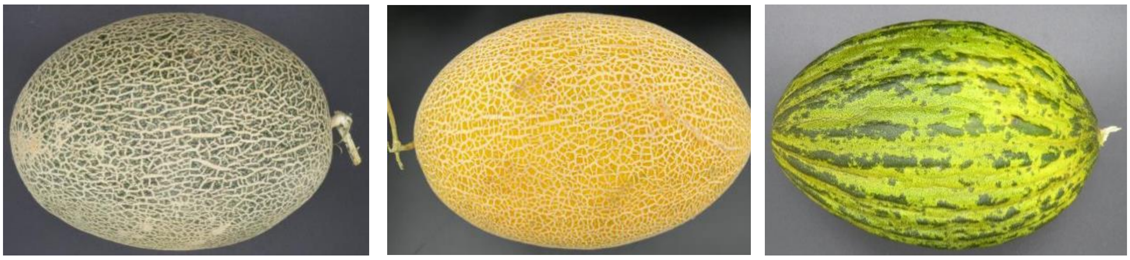

A B C

Figure S1. Three Hami melon landraces (A: 'Xizhoumi 17'; B: 'Jinhuami 25'; C: 'Chougua') from Xinjiang Province, China used in this study.
